# The Flexibility of Nonconsciously Deployed Cognitive Processes: Evidence from Masked Congruence Priming

**DOI:** 10.1371/journal.pone.0017095

**Published:** 2011-02-10

**Authors:** Matthew Finkbeiner, Jason Friedman

**Affiliations:** Macquarie Centre for Cognitive Science, Macquarie University, Sydney, New South Wales, Australia; National Institute of Mental Health, United States of America

## Abstract

**Background:**

It is well accepted in the subliminal priming literature that task-level properties modulate nonconscious processes. For example, in tasks with a limited number of targets, subliminal priming effects are limited to primes that are physically similar to the targets. In contrast, when a large number of targets are used, subliminal priming effects are observed for primes that share a semantic (but not necessarily physical) relationship with the target. Findings such as these have led researchers to conclude that task-level properties can direct nonconscious processes to be deployed exclusively over central (semantic) or peripheral (physically specified) representations.

**Principal Findings:**

We find distinct patterns of masked priming for “novel” and “repeated” primes within a single task context. Novel primes never appear as targets and thus are not seen consciously in the experiment. Repeated primes do appear as targets, thereby lending themselves to the establishment of peripheral stimulus-response mappings. If the source of the masked priming effect were exclusively central or peripheral, then both novel and repeated primes should yield similar patterns of priming. In contrast, we find that both novel and repeated primes produce robust, yet distinct, patterns of priming.

**Conclusions:**

Our findings indicate that nonconsciously elicited cognitive processes can be flexibly deployed over both central and peripheral representations within a single task context. While we agree that task-level properties can influence nonconscious processes, our findings sharply constrain the extent of this influence. Specifically, our findings are inconsistent with extant accounts which hold that the influence of task-level properties is strong enough to restrict the deployment of nonconsciously elicited cognitive processes to a single type of representation (i.e. central *or* peripheral).

## Introduction

Research on the nonconscious human mind has progressed in fits and starts. Prior to the late 1990s, claims of nonconscious perception were met with strong criticism and were invariably undermined by subsequent research. But the failures of early attempts were primarily due to methodological problems that have been addressed in recent years (cf. [1]–[2] for reviews). Over the last two decades, researchers have convincingly demonstrated that subliminally presented stimuli influence both behavioral measures such as reaction times or accuracy rates [3]–[7] and neurophysiological measures such as electrical brain activity (event-related potentials) [8], [9] and cerebral blood flow (functional MRI) [10]–[14]. Nevertheless, despite the widespread agreement that nonconscious perception is an area amenable to rigorous scientific investigation, there is still little consensus amongst current theories as to how subliminally presented stimuli exert their influence over an individual's behavior. Broadly speaking, there presently are two competing views: one which posits that nonconscious processes engage central mental representations [15]–[18], and one which posits that they are limited to peripheral representations [19]–[23]. Note that we borrow the terms “central” and “peripheral” from Turvey [24], who used the term “peripheral” to describe the representations and processes that are engendered and carried out in the earliest (sensory) stages of visual processing. As such, a peripheral representation is specified in terms of its corresponding stimulus' physical properties. In contrast, the term “central” is used to describe the representations and processes that are accessed and carried out in later stages of information processing, where the content of processing has been abstracted away from the physical stimulus. Importantly, while empirical support has been provided for both the central and peripheral accounts of nonconscious processes from a range of different tasks, there has not yet been a convincing falsification of either position. Given the failure to reject either position (despite vigorous attempts from both sides), it is somewhat surprising that a third position, which holds that subliminal effects arise from *both* central and peripheral sources, has not been put forth. The purpose of the present article is to provide evidence in support of this third position.

### The masked congruence effect

The seminal work within the modern era on subliminal priming appeared in 1998 by Dehaene and colleagues [16]. They found that subliminal number primes modulated both behavioral and neurophysiological measures in a number magnitude-judgment task. In this now widely-used task, subjects are asked to categorize the target numbers 1 through 9 (excluding 5) as “larger or smaller than five”. Critically, the targets are preceded by a heavily masked prime stimulus that is either congruent with the target (i.e. both prime and target fall on the same side of 5) or incongruent (prime and target fall on opposite sides of 5). In this paradigm, subjects respond faster and more accurately in the congruent condition than they do in the incongruent condition, despite not being able to detect the presence of the prime stimuli. This effect is known as the *masked congruence effect* (MCE) and it is very robust, having been observed with a wide range of stimulus types and in many different laboratories.

According to Dehaene and colleagues [15]–[16], [25]–[26], the MCE is the result of subjects applying the task instructions to the masked prime. On this view, subjects consciously prepare a ‘chain of processes’ on the basis of the task instructions and then nonconsciously apply this chain of processes to the subliminal primes. This interpretation was motivated by their neurophysiological findings which revealed that the masked number primes modulated electrical and hemodynamic activity over the motor cortices. Specifically, Dehaene et al. [16] found that the lateralized readiness potential (LRP) and the “lateralized bold response” revealed increased brain activity over the ipsilateral cortex when the targets were preceded by incongruent primes. In other words, subjects were processing the prime stimuli all the way up to the point of preparing a (covert) response to the subliminal prime. We recently extended these findings in a pointing paradigm by showing that the congruence of the subliminally presented prime systematically shaped *overt* reaching responses [27]. In sum, it is clear now that the processing of subliminally presented primes is not restricted to early visual areas but extends up to include the preparation of a task-appropriate motor response.

When Dehaene et al. [16] first suggested that subjects could apply a consciously prepared chain of processes to a nonconsciously presented prime, there was a great deal of skepticism because this notion sharply contradicted the long-standing belief that nonconscious cognitive processes were carried out independently of conscious strategies. In the meantime, though, there have been many findings reported that are consistent with Dehaene et al.'s proposal and nowadays it is widely accepted in the masked priming literature that higher-level cognitive systems or resources (e.g. attention and expectation) can modulate the processing of subliminally presented stimuli [1], [8], [25], [28]–[34].

### Do nonconscious processes engage central or peripheral representations?

A far more controversial claim by Dehaene et al. [16], and one that continues to be vigorously debated, holds that the ‘chain of processes’ applied to subliminal primes operate over central representations. Findings consistent with this possibility have come from a range of different studies and research groups [5], [17]–[18], [26], [29], [33], [35]–[41], but certainly not without challenges. Perhaps the best known challenge came from Damian [19], who argued that the MCE observed by Dehaene et al. was due to the conflict that arose between learned stimulus-response (SR) mappings, not semantic categories. Damian observed that masked congruent primes produced a robust MCE, but only for “repeated” primes – i.e. primes that had appeared as targets in the experiment and thus had elicited overt responses. In contrast, so-called “novel” primes, which had not appeared as targets and were thus never perceived consciously in the experiment, did not produce any priming. Damian took these findings to suggest that the MCE observed by Dehaene et al. was best understood in terms of the *direct motor specification* hypothesis [42], according to which subliminal priming effects reflect the nonconscious triggering of a motor response that has come to be associated with a specific physical stimulus. Since Damian's very influential paper, researchers have been careful to use novel primes in masked congruence priming experiments and, contrary to Damian's conclusions, there are now several reports of novel primes producing robust subliminal priming effects. The critical difference between Damian's original study and recent studies that report priming with novel primes appears to hinge on the use of typical exemplars from well defined semantic categories. Damian asked his subjects to indicate if a word's referent was bigger or smaller than a 20 cm×20 cm square depicted on the computer monitor. Contrast this with a task in which subjects are asked, for example, to indicate whether “lion” is an animal or a vegetable. The latter task employs typical exemplars (lion) of well defined semantic categories (animal, vegetable), whereas Damian's task used ad hoc categories (bigger or smaller than a 20 cm square).

From 2001, when Damian's paper appeared, a considerable amount of time and effort has been spent on trying to adjudicate between the central and peripheral accounts of subliminal priming effects [2]. Presently, it appears that one reason for the long-running debate is that the depth of processing of subliminal primes depends upon many factors, including stimulus- and experiment-level variables. For example, it has been found in recent studies that the number of targets that are used in the experiment (i.e. target set size) can determine whether an MCE is obtained for novel primes (large set size) or only for repeated primes (small set size) [34], [38], [43]. Thus, in some experiments it appears that the MCE stems from the overlap in processing of central representations, while in other experiments the MCE appears to stem from learned associations between specific physical forms and their appropriate responses. This apparent dichotomy raises an interesting question. Namely, is there a principled reason to think that the “chain of processes” applied to subliminal primes must *either* involve central processes or not? Or is it reasonable to think that nonconsciously engaged processes can be applied flexibly such that they could operate over *both* central and peripheral representations?

Previous investigations of the depth of processing of subliminal primes have tried to adjudicate between the central and peripheral accounts of subliminal priming by creating experimental contexts that reveal one type of priming or the other. Our approach is different because our research question is different. While it is now very clear that the task conditions can serve to restrict the range or depth of nonconsciously recruited cognitive processes, we are not convinced that subliminal priming effects must *in principle* be exclusive such that they are either central or peripheral. Rather, it strikes us as more reasonable to posit an inclusive hypothesis whereby nonconsciously deployed cognitive processes are, by default, flexible enough to be applied over both central and peripheral representations. Thus, in the present study we pursue the possibility that the same experimental context can yield evidence for both central and peripheral sources of subliminal priming. In the experiments below, we demonstrate just this. For the first time that we are aware of, we show that a single experimental context can produce an MCE that is comprised of both central and peripheral sources.

## Results

The purpose of this project was to investigate the depth of processing of nonconsciously perceived prime stimuli within a single experimental context. We chose to use subjects' reaching trajectories as our dependent measure [27], [44] because of our interest in the temporal dynamics of the masked congruence effect. Reaching trajectories are unique in that they allow one to analyze the shapes of the actual trajectories (which can vary by experimental condition) as well as ascertaining the properties of the planned movements (submovements), some of which are only partially executed. The advantage of analyzing submovements comes from their discrete (ballistic) properties. That is, because submovements are not updated during execution (although a new submovement may commence before the previous one has completed), it is possible to identify the participant's intended movement at the onset of the submovement, which allows much earlier access to the state of the decision making process. A second reason for using reaching trajectories as our dependent measure came from a pilot experiment that revealed distinct MCEs for novel and repeated primes. This was a surprising finding because results from standard reaction time studies have yielded largely indistinguishable MCEs for novel and repeated primes within the same experimental context [26]. To ensure that our findings could be replicated, we have repeated our initial pilot study several times with several different stimulus types and in each case we have found that repeated primes produce an earlier and larger MCE than novel primes [45]. Here we report two critical experiments from this series of experiments.

### Experiment 1: Animal/Person Categorization

**Figure 1:**
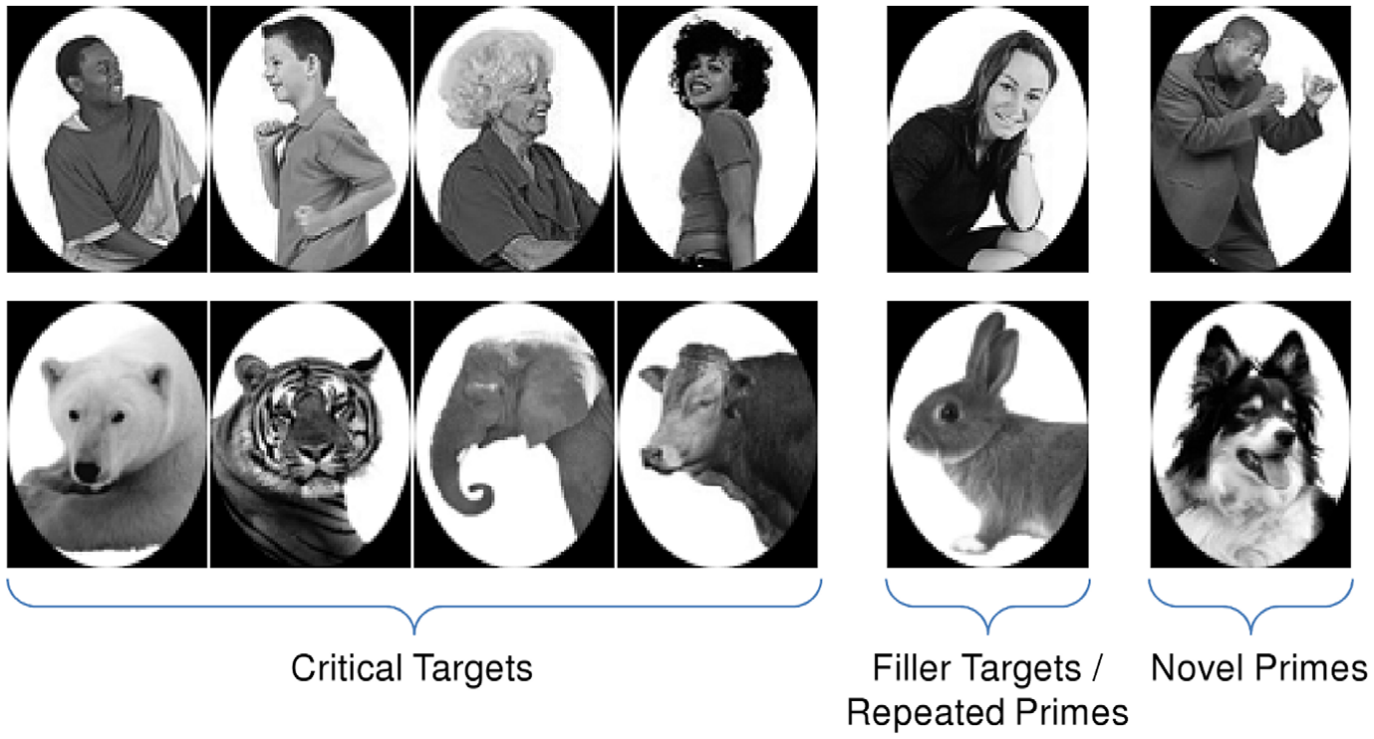
Stimuli used in Experiment 1.

**Figure 2:**
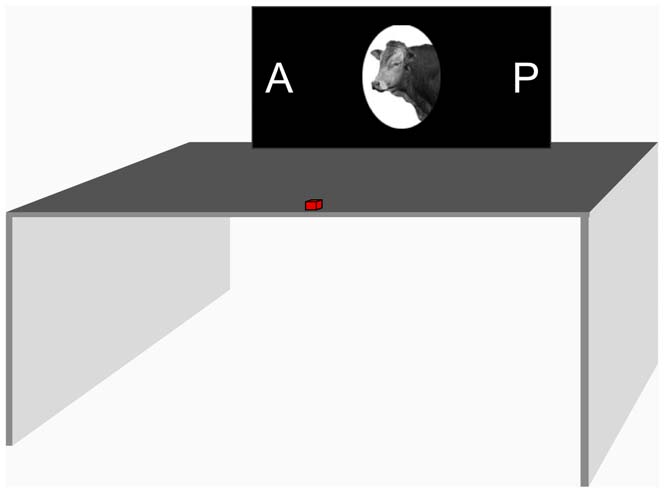
Experimental Apparatus. Subjects initiated trials by pressing the red button. They then responded by reaching out and touching the ‘A’ for animal targets or the ‘P’ for person targets.

**Figure 3:**
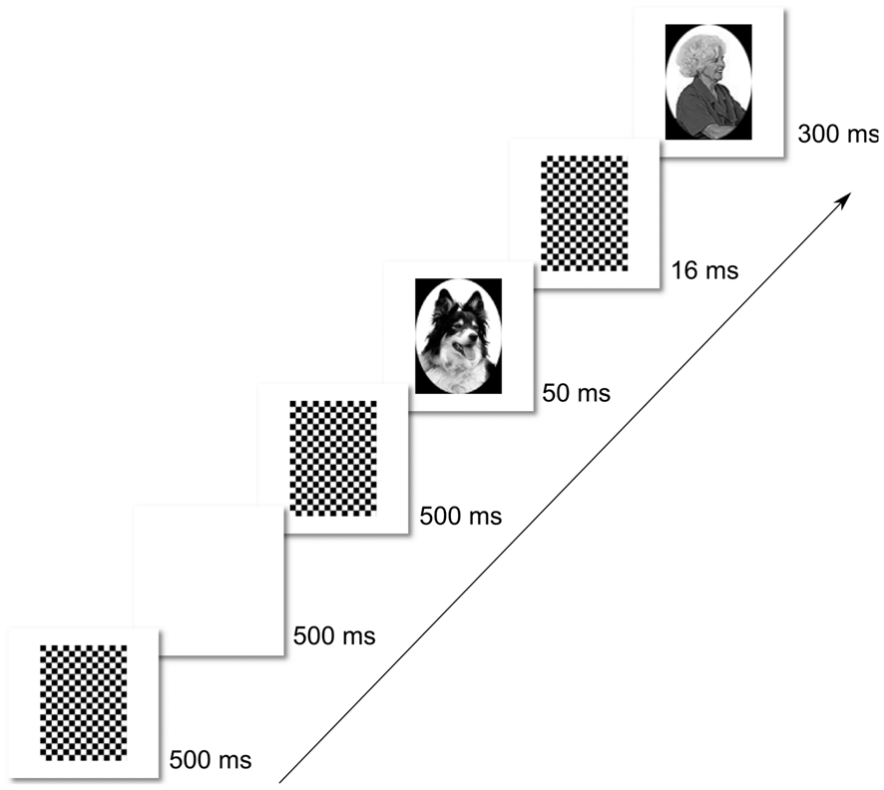
Trial Structure in Experiment 1. Here we depict an incongruent trial, in which a Person target follows the Animal prime stimulus. The forward and backward masks are complementary checkerboard patterns. The prime duration is 50 ms; the target duration is 300 ms.

In Experiment 1, subjects categorized pictures of people or animals (see Figure 1 for all stimuli) by reaching out and touching a “P” to indicate person or an “A” to indicate animal (see Figure 2 for a schematic of the experimental apparatus). Eight of the targets (4 animals and 4 people) were designated as critical targets and the two remaining targets were designated as filler targets. All targets appeared equally often, but the filler targets also appeared as subliminal (repeated) primes. We ensured that the novel and repeated prime stimuli appeared equally often across the experiment (see methods); we also counterbalanced the novel and repeated primes across subjects (N = 16). Thus, across the experiment, the only difference between the repeated and novel primes was that each repeated prime appeared as the target on 10% of the trials. The trial structure we used is very common in masked priming experiments and is depicted in Figure 3.

**Figure 4:**
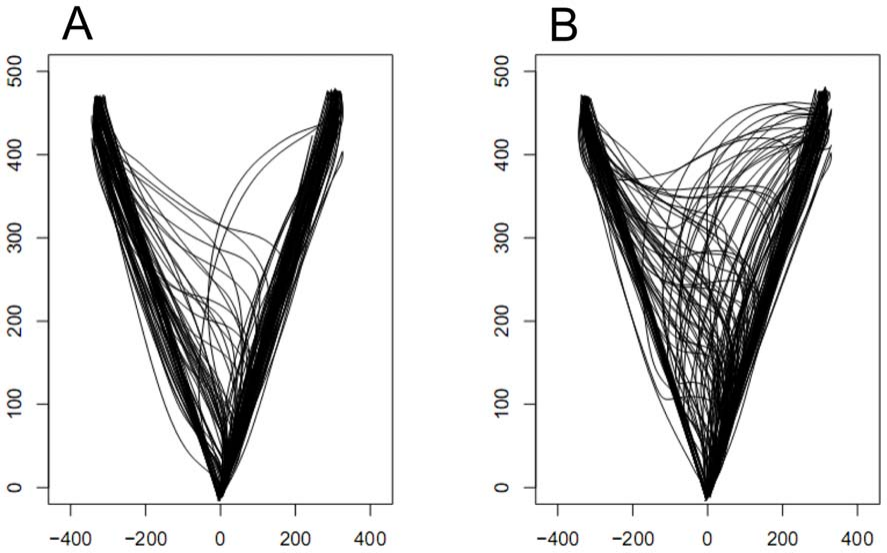
Sample data from one subject in Experiment 1. Panel A depicts reaching trajectories in the congruent condition; Panel B depicts reaching trajectories in the incongruent condition.

From the pointing data, we extracted two dependent measures. First, we calculated the *path offset value*. This value is calculated by first measuring the distance (D) of the straight line between the start and end points of the trajectory. Then, at each time point from target onset (sampled every 5 ms), the shortest distance between the tip of the index finger and the straight line is calculated (pD). Finally, the path offset value is taken as the ratio of the two distances (pD/D). The larger this ratio, the more “wayward” the finger's flight. As we have reported previously [27], [45], and as is clear in Figure 4, subjects' responses are often initially governed by the masked primes. This influence of the masked prime on the finger's initial flight path produces larger path offset values in the incongruent condition than in the congruent condition. The MCE is calculated by subtracting, at each time point, each subject's mean path offset value in the congruent condition from the corresponding mean value in the incongruent condition. The question of interest in the present experiment was whether both novel and repeated primes produced a masked congruence effect and whether the MCE for one prime type was distinct from the other.

**Figure 5:**
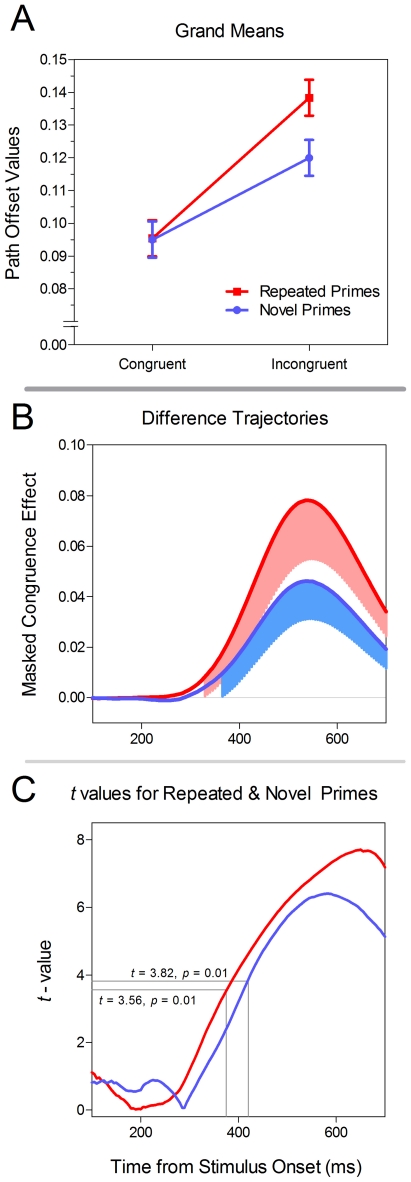
Results from Experiment 1. In Panel A, the grand means by Prime Type and Prime Congruence are depicted. In Panel B, the difference trajectories (incongruent – congruent) are depicted for each Prime Type. The shaded regions indicate 95% confidence intervals that do not include zero (i.e. a significant MCE). Panel C depicts the observed *t*-values for each Prime Type. The critical *t*-values are indicated with grey lines.

Replicating previous findings [26], we found that both novel and repeated primes produced a significant MCE. Unique to this study was our finding of an interaction between Prime Type (repeated vs. novel) and Prime Congruence (congruent vs. incongruent) such that repeated primes produced an earlier and larger MCE than novel primes. Here we report three different analyses with the path offset measure that compel this conclusion. First, we calculated the conditional means across the average path offset values between 300 and 550 ms after target onset (see Figure 5A) and entered them into a 2×2 repeated measures ANOVA with the factors Prime Type and Prime Congruence. This analysis of ‘grand means’ revealed main effects of Prime Type (*F*(1,15) = 7.5, *p* = 0.01) and Prime Congruence (*F*(1,15) = 34.5, *p*<0.01), as well as a reliable interaction between the two factors (*F*(1,15) = 6.4, *p* = 0.02).

Second, to better understand the time course of the main effects as well as the interaction, we conducted the same 2×2 ANOVA at each time point from 100 to 700 ms from target onset (see Song and Nakayama [46] for a similar procedure). This series of ANOVAs revealed significant main effects of Prime Type (*p*<0.05, uncorrected) between 415 ms and 630 ms, and Prime Congruence from 330 ms until the end of the epoch. The interaction between the two factors was reliable from 415 ms until the end of the epoch. To facilitate visualization of these results over time, we have plotted the mean difference scores (incongruent – congruent) in Figure 5B. The shaded regions indicate the 95% confidence intervals that do not include zero (i.e. the regions for which the path offset values in the incongruent condition were significantly greater than in the congruent condition).

Third, to control for the multiple comparisons that are required in the analysis of continuous data, we used a permutation procedure described by Blair and Karniski [47] to produce a reference distribution of *maximum t*-values (N = 2^16^). We set our critical *t*-value equal to the value in the reference distribution that corresponded to the 99^th^ percentile. The advantage of this procedure for continuous data is that it allows the researcher to maintain experimentwise error at a prescribed level (we used 0.01) whereas alternative methods (e.g. Bonferroni correction) simply ensure that a desired level is not exceeded. The details of this analysis are provided below in the methods section. The critical *t*-values were 3.56 and 3.82 for the repeated and novel primes respectively. The observed t-values exceeded these cutoffs beginning at 375 ms with repeated primes and at 420 ms with novel primes (see Figure 5C).

**Figure 6:**
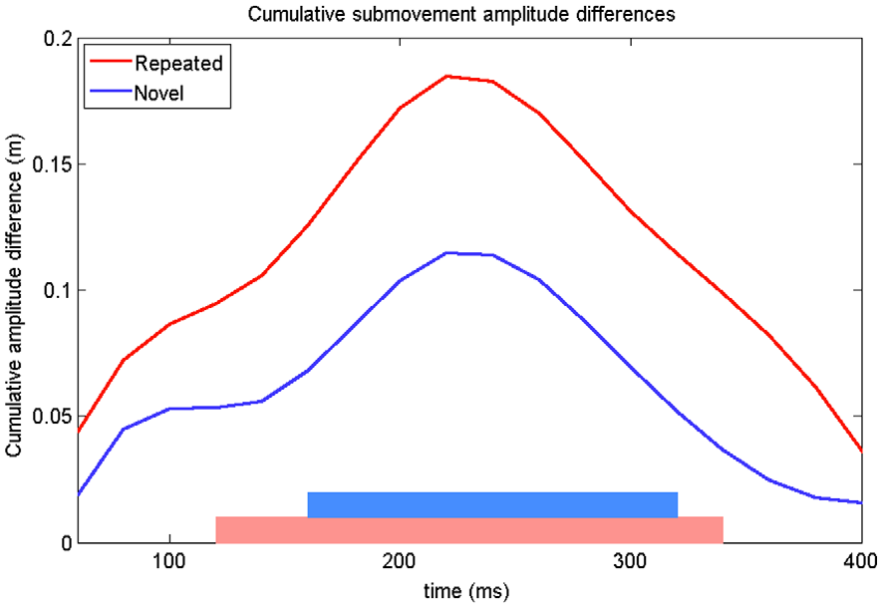
Submovement cumulative amplitude differences from Experiment 1. The difference (incongruent – congruent) in the mean cumulative submovement amplitude is plotted. The shaded regions along the x-axis indicate when the 95% confidence intervals calculated over the difference scores do not include zero.

Finally, we used a second dependent measure, *cumulative submovement amplitude*, to better determine the temporal properties of the MCE for novel and repeated primes. As this is a measure of intent, it provides an earlier window into the decision making process than the path offset measure. Cumulative submovement amplitude is a measure of how far the subject is *planning* on going towards (or away from) the target. Figure 6 shows the differences in mean (across subjects) cumulative submovement amplitudes between the congruent and incongruent trials. As observed in the path offset analysis, the repeated trials showed an earlier and larger MCE than the novel trials. For the repeated prime stimuli, this difference was significantly greater than zero (using a t-test with 0.05 significance) between 120 ms and 340 ms, while for the novel prime stimuli, this was between 160 ms and 320 ms.

The test of prime visibility (see Methods for details) revealed that the masking procedure was effective in preventing visual awareness of the prime stimuli. The mean hit rate was 47% and the mean false alarm rate was 49.5%, which yielded a d' of −0.04. A one-sample t-test indicated that this d' score was not different from zero (*t*(15) = −0.8, *p* = 0.43). A further analysis was used to assess prime visibility for repeated primes and novel primes independently. This analysis revealed d' scores of −0.07 and −0.009 for repeated and novel primes respectively; neither score was different from zero (both *t*-values <1).

### Experiment 2: Letter/Number Categorization

The results of Experiment 1 revealed a clear difference between repeated and novel primes, with repeated primes producing an earlier and larger MCE than novel primes. Because the frequency of occurrence for each prime type was held constant, and because we counterbalanced the assignment of prime stimuli to each prime type across subjects, the only difference between the two was that repeated primes were available for conscious evaluation and novel primes were not. If one wanted to maintain that our experimental conditions led to a central source of priming (which the MCE for novel primes suggests), thereby excluding a peripheral source, then one would need to argue that the earlier and larger priming effect for the repeated primes was due to a consciously induced modulation of the central representations for the repeated stimuli. In Experiment 2 we tested this possibility against the alternative possibility that conscious evaluation of stimuli leads subjects to establish peripheral stimulus-response associations and that these SR mappings were responsible for the earlier MCE.

In Experiment 2 subjects categorized strings of letters or numbers by reaching out and touching “ABC” on one side of the computer monitor or “123” on the other side. We used this task because the processing of letters and digits proceeds through well-established and clearly delineated stages. Initially, for example, letters are processed as non-letter-specific shapes in both hemispheres, followed by the retrieval of letter-specific representations in the left hemisphere [48]–[50]. Letter-specific representations are commonly referred to as abstract letter identities, or ALIs, because they serve to mediate between highly variable inputs (consider the variability in handwritten text) and lexical orthographic representations stored in long-term memory. Importantly for our purposes here, there is a long line of evidence, both from studies of neuropsychological patients and unimpaired individuals, that ALIs are obligatorily accessed independently of a letter-string's physical properties, such as its shape, size or location [51].

To isolate peripheral and central sources of the MCE, we compared priming for stimuli like “EhT” versus “eHt”. Both stimuli map onto the same abstract letter identities, but we ensured that only one of the two physical forms appeared consciously as a target during the course of the experiment. The target stimulus in this pair also served as the repeated prime. The other stimulus in this pair, which we refer to as the “repeated/novel” prime, is both “repeated” (it is identical to its pair at the level of abstract letter identities) and “novel” (its physical form never appears consciously). Critically, if the distinctive MCE for repeated primes in Experiment 1 was due to an exclusive modulation of central representations, then repeated and repeated/novel primes should produce the same pattern priming in the present experiment by virtue of sharing identical abstract letter identities. If, though, the distinctive MCE for repeated primes in Experiment 1 was due to a learned association between a particular physical form and its appropriate response, then the MCE for repeated primes in this experiment should be distinct from the MCE for novel and repeated/novel primes.

**Figure 7:**
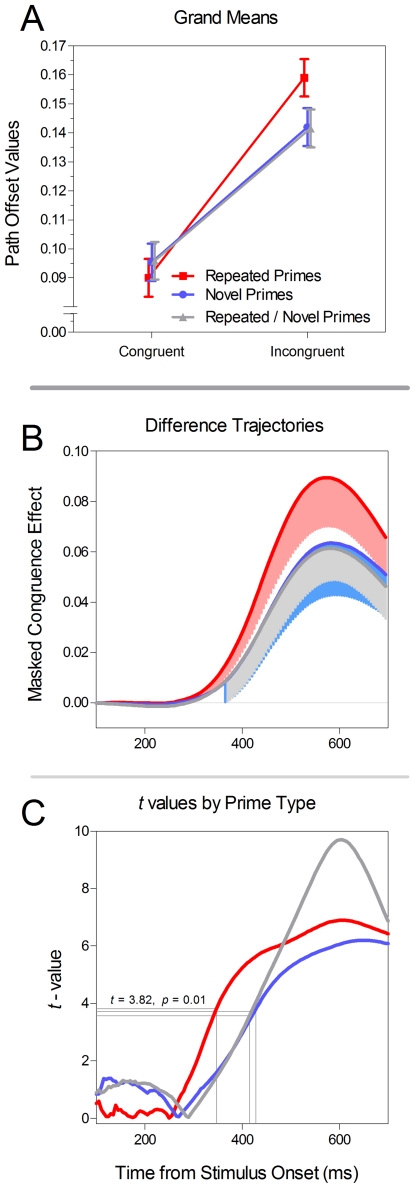
Results from Experiment 2. In Panel A, the grand means by Prime Type and Prime Congruence are depicted. In Panel B, the difference trajectories (incongruent – congruent) are depicted for each Prime Type. The shaded regions indicate 95% confidence intervals that do not include zero (i.e. a significant MCE). Panel C depicts the observed *t*-values for each Prime Type. The critical *t*-values are depicted by grey lines.

Except for the obvious change in the task and the use of three prime types (repeated, novel and repeated/novel), the two experiments were identical. Just as before, we calculated the ‘grand means’ between 300 and 550 ms after target onset (see Figure 7A) and entered them into a 3×2 repeated measures ANOVA with the factors Prime Type and Prime Congruence. This analysis revealed a main effect of Prime Congruence (*F*(1,15) = 56.4, *p*<0.01), no effect of Prime Type (*F*(2,30) = 1.85, *p*>0.05), but a reliable interaction between the two factors (*F*(2,30) = 4.3, *p* = 0.02).

We again conducted the same 3×2 ANOVA at each time point (100–700 ms from target onset). Similar to the analysis of the grand means above, there was no effect of Prime Type, but the effect of Congruence was significant (*p*<0.05, uncorrected) beginning at 330 ms and continuing through the end of the epoch. The interaction was reliable between 415 ms and 600 ms. Again, to facilitate visualization of these results over time, the difference trajectories are presented in Figure 7B.

Once again, to control for the multiple comparisons, we made use of the permutation procedure mentioned above. As is clear in Figure 7C, each prime type produced a robust MCE with critical *t*-values established at 3.82, 3.74 and 3.57 for the repeated, novel and repeated/novel primes respectively. The observed t-values exceeded these cutoffs beginning at 350 ms with repeated primes, 425 ms with novel primes and 415 ms with the repeated/novel primes (see Figure 7C). Critically, it is clear in Figure 7 that the MCE for repeated primes patterned very distinctly relative to the other two prime types. Likewise, the repeated/novel primes patterned very similarly to the novel primes, both in terms of magnitude of priming (Panels A & B) and in the time course of priming (Panel C).

**Figure 8:**
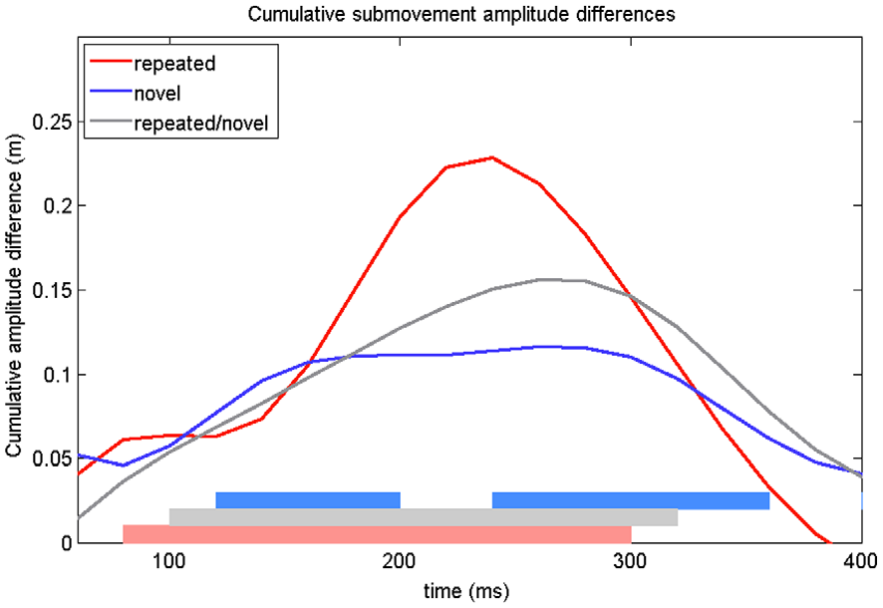
Submovement cumulative amplitude differences from Experiment 2. The difference (incongruent – congruent) in the mean cumulative submovement amplitude is plotted. The shaded regions along the x-axis indicate when the 95% confidence intervals calculated over the difference scores do not include zero.

The differences in cumulative submovement amplitude were calculated as in Experiment 1, and are shown in Figure 8. Similar to Experiment 1, the repeated primes produced an earlier MCE (60 ms to 300 ms) than the novel primes (120 to 200 ms, and then 240 to 360 ms). The repeated/novel primes produced a significant MCE from 100 to 320 ms.

The test of prime visibility again revealed that the masking procedure was effective in preventing visual awareness. The mean hit rate was 51.7% and the mean false alarm rate was 48.3%, which yielded a d' score of 0.06. A one-sample t-test indicated that this d' score was not significantly different from zero (*t*(15) = 1.06). An assessment of prime visibility for each of the prime types independently revealed d' scores of 0.09, −0.01 and 0.10 for the repeated, novel and repeated/novel primes respectively; no score was different from the null mean (all *t*-values <1.1).

## Discussion

The research reported here establishes the new and important result that novel and repeated primes yield distinct patterns of subliminal priming within the same experimental context. Specifically, repeated primes produce an earlier and larger masked congruence effect (MCE) than novel primes. This finding is very robust, having been replicated in the two experiments reported here as well as in others from our lab [45]. The earlier and larger MCE for repeated primes is not due to differences between the specific stimuli that were assigned to each prime type because stimulus assignment was counterbalanced across subjects. Likewise, the difference in the MCEs is not due to differences in frequency of occurrence because novel and repeated primes appeared equally often in each block of the experiment. The only difference between novel and repeated primes was that repeated primes appeared as targets on a small proportion of trials (10% in Experiment 1 and 5% in Experiment 2). Thus, it seems reasonable to attribute the difference in the MCE to the fact that repeated primes were consciously evaluated and overtly responded to on a proportion of trials, whereas novel primes were not. How might consciously evaluating and responding to a stimulus lead to differences in the time course and magnitude of the masked congruence effect? We consider two possibilities here.

On the first possibility, which we term the *exclusive* possibility, the MCE is due to a chain of cognitive processes being deployed nonconsciously over either central or peripheral representations as a function of the task demands or target set size [34], [43]. On this possibility, the MCE that we observed in our study with novel primes would compel the conclusion that the MCE in our task stemmed from the overlap in processing of central representations. Pursuing this possibility a bit further, one would need to conclude that the earlier and larger MCE that we observed with repeated primes was due to changes in the central representations (or their connections) that corresponded to the repeated prime stimuli. Critically, the argument would continue that these representational changes were brought on by the conscious appreciation of the repeated stimuli, thereby ensuring that the changes were selective for the repeated stimuli. While we find this line of reasoning to be both logically possible and plausible, it is undermined by the results of our Experiment 2.

If the differences in the MCEs for repeated and novel primes were due to consciously-induced changes in the central representations that corresponded to the repeated stimuli, then two physically different stimuli (e.g. “EhT” versus “eHt”) that map onto the same abstract letter identities should produce an equivalent MCE. We tested this possibility in Experiment 2 and found that the MCE for “repeated/novel” primes (i.e. primes that shared identical abstract letter identities with the repeated primes but whose physical form was novel) was very similar to the MCE for novel primes (i.e. primes that were novel at both the level of abstract letter identities and physical form). The MCE for the repeated primes was both larger and earlier than the MCEs for the novel and repeated/novel primes. Thus it seems unlikely that the difference between the MCE for the repeated primes on one hand and the MCEs for the novel and repeated/novel primes on the other can be attributed to modulations within a single central source.

On the second possibility that we consider, which we term the *inclusive* possibility, the MCE is due to a chain of cognitive processes being flexibly deployed over both central and peripheral representations. On this possibility, different properties of the task can serve to modulate the extent to which the representations of one kind or another are considered in the stimulus evaluation process. This is what is meant by “flexibly deployed”. Thus, while the inclusive hypothesis does not deny the possible influence of task-level properties on the deployment of nonconscious processes, this hypothesis allows for an MCE from multiple sources within a single experimental context whereas the exclusive hypothesis does not. The findings reported above, especially those of Experiment 2, are consistent with the inclusive hypothesis but inconsistent with the exclusive hypothesis.

Because the inclusive hypothesis posits that nonconscious cognitive processes may be deployed over more than one type of representation, it follows from this hypothesis that primes which map onto multiple types of representations should produce earlier (and perhaps larger) priming effects than primes which only map onto a single representation type. The rationale is straightforward. Due to noise within the information processing system, the time that a particular mental representation is accessed will vary from trial to trial, with access occurring early on some trials and relatively late on others. On the assumption that the distributions of access times are similar for both types of representations, and that the representation accessed first is the one that governs (at least initially) the response (e.g. “Animal” or “Person”), it follows that the response, and by extension the MCE, will be influenced earlier when sampling from two distributions than when sampling from one distribution. In our particular case, we suggest that the MCE arises earlier in the case of repeated primes than novel primes because repeated primes map onto two independent types of representations – one central and one peripheral – each of which is associated with a separate distribution of access times. Novel primes, on the other hand, are not associated with an SR mapping and, hence, must map onto their appropriate responses indirectly via a central representation. Another possibility is that peripheral SR mappings engender a response more quickly than mappings via a central route.

### An alternative non-semantic account

The skeptic of nonconscious processes engaging central representations will prefer to explain our findings in terms of an account that appeals to peripheral SR mappings for both novel and repeated prime types. On this peripheral account, the larger MCE for repeated primes would be attributed to better established SR mappings by virtue of these stimuli having been available for conscious evaluation. But how does this alternative account explain priming for the novel and repeated/novel primes? These stimuli never appeared consciously, which is a widely-assumed prerequisite for the establishment of SR mappings. A variant of the peripheral account that gets around this problem is the ‘action-trigger’ account [20]. On this account, the perceptual features of *expected* stimuli can enter into an SR mapping despite these stimuli never being available for conscious evaluation and learning. This account is plausible in the context of very small categories (e.g. the numbers 1–9), but it becomes untenable with large categories because it is unlikely that an individual can ‘expect’ all of the perceptual features that are needed to compile the full range of exemplars in these categories. And it is worth noting that the categories in the experiments reported above were very large. In Experiment 1, the categories were ANIMAL or PERSON, both of which are comprised of a very large number of perceptually complex exemplars. In Experiment 2, the categories were LETTER or NUMBER. In this latter case, if we had used a single font, these categories would have been comprised of 26 exemplars in the case of letters and 10 exemplars in the case of numbers. But in our experiment each letter and number in each 3-item string was printed in one of 13 different fonts, thereby requiring individuals to prepare 38,614,472 different percepts of the possible 3-letter letter strings and 2,197,000 different percepts of the possible 3-digit number strings. To be clear, in the case of letters, there were 26 possible letters in each of 13 different font faces (26*13) in each of the 3 positions (because repetition was allowed). The total number of possible 3-letter strings then was (26*13)^3^ = 38,614,472. In the case of the digit strings, there were 130 possible forms at each position (10 digits and 13 fonts), yielding 2,197,000 possible 3-digit strings (10*13)^3^.

While preparing a percept of the full 3-item string seems extremely unlikely given the range of possible forms, it is also unlikely that individuals use lower-level perceptual features (e.g. ‘curved line’). This is because it is hard to imagine what, if any, perceptual features uniquely identify letters or numbers. For example, the feature “curved line at the top” might be a “Q” or an “S” or a “C”, but it might also be a “0” or an “8” or a “9”. Similarly, “straight vertical line” might be a “1” but it could also be an “L” or an “F”. And so on. Thus, it seems that the only ‘expected features’ that one could employ to do the letter/number task are not features at all but, rather, abstract letter or number identities. But of course, if one were to re-formulate the ‘action trigger’ account to include abstract-level triggers, as some have done [43], [52], we would be left with an exclusively central source of the MCE. And importantly for our purposes here, the results reported above (especially from Experiment 2) are inconsistent with an account that posits an exclusively central source.

### Conclusion

The results reported in our study firmly establish that the nonconscious processing of two different stimulus types (in this case novel and repeated prime stimuli) can yield distinct masked congruence effects within the same experimental context. Our findings are inconsistent with extant ‘exclusive’ hypotheses which posit that the task demands direct nonconsciously elicited cognitive processes to be deployed over *either* central representations *or* peripheral representations. These accounts fail to predict the difference in magnitude and time course of the masked congruence effect for novel and repeated subliminal primes within the same experimental context. To provide an adequate explanation of how nonconsciously elicited processes could yield distinct patterns of behavior within a single task, we considered a new ‘inclusive’ hypothesis. From this perspective, the scope of nonconsciously elicited cognitive processes is widened to include both central and peripheral representations. With this possibility in mind, we hypothesized that stimuli which can be processed both centrally and peripherally (e.g. repeated prime stimuli) would yield earlier priming effects than stimuli that can only be processed centrally (e.g. novel prime stimuli). Our findings confirm this hypothesis by revealing an earlier and larger masked congruence effect for repeated primes than novel primes.

## Methods

### Ethics Statement

The ethical aspects of this study were approved by the Macquarie University Ethics Review Committee (Human Research).

### Experiment 1: Animal/Person Categorization

#### Subjects

Sixteen individuals participated for course credit in an introductory psychology course. All individuals were right handed and gave informed written consent in accord with Macquarie University Human Research Ethics. The masking procedure was not effective with 4 subjects and these individuals were replaced (see below). Thus a total of 20 individuals were tested.

#### Materials and Design

The stimuli used in the experiment are depicted in Figure 1. The critical targets (N = 8) were comprised of 4 person pictures and 4 animal pictures. In each block of 40 trials, the critical targets were presented 4 times, once with each prime type (novel congruent, novel incongruent, repeated congruent, repeated incongruent), for a total of 32 critical (analyzed) trials per block. Thus the novel and repeated prime stimuli appeared equally often as masked primes across the 32 critical trials in each block. Additionally, each block included 8 filler trials. The targets on the filler trials were the repeated primes (4 instances of the person filler target and 4 instances of the animal filler target). The prime stimuli on the filler trials were selected from the set of novel primes and were equally distributed across the 8 filler trials. Thus in the filler trials the repeated primes appeared 4 times each (as targets) and the novel primes appeared 4 times each (as masked primes). We used this procedure to equate the frequency of occurrence of the novel and repeated primes across each block of trials. To ensure that any observed differences between novel and repeated primes were not due to the particular stimuli, the assignment of the specific stimuli to each prime type was counterbalanced across subjects. Thus, across the experiment, the only difference between novel and repeated stimuli was that each repeated prime stimulus appeared as a visible target on 10% of the trials.

#### Procedure

Subjects sat at a table with a touch screen LCD monitor fixed in position 50 cm from the edge of the table (see Figure 2). Throughout the experiment, the letter ‘A’ appeared on one side of the monitor and the letter ‘P’ appeared on the other side. Subjects responded by reaching out and touching the A for animal pictures and the P for person pictures. The position of the A and P was counterbalanced across subjects. Subjects initiated each trial by pressing a button that was aligned with the body midline and positioned 5 cm from the front edge of the table. To encourage subjects to respond very quickly, they were required to release the button and begin the forward movement of their response within 350 ms of target onset. Failure to do this resulted in the trial being aborted, with aborted trials being repeated at the end of the block. Upon initiating a trial, subjects had 4 seconds to register a response by touching the monitor. An Optotrak Certus with a 200 Hz sampling rate was used to track the position of a small light-emitting diode that was affixed to the tip of the right index finger. The trial structure is depicted in Figure 3. Each event within the trial followed immediately after the preceding event with no intervening video frames. The stimulus display was controlled with Presentation software (Neurobehavioral Systems) and custom software written to interface with the Optotrak Certus.

The experiment included 2 practice blocks (80 trials) that were not analyzed, 10 experimental blocks (400 trials) and 2 prime detection blocks (80 trials). The trials in all blocks were identical except that in the detection blocks subjects were informed of the presence of the primes and on each trial the question “Was the prime an animal or a person?” appeared following target offset. Subjects indicated their response by pressing one of two buttons. To emphasize the importance of accuracy in this task, and to guard against the prime detection measure from being contaminated by nonconscious processes, the cue to respond in the detection task was delayed for 1 second following the offset of the target stimulus.

#### Analysis

Path Offsets. The position of the index finger was sampled every 5 ms beginning with target onset and continuing until a response was registered. To determine the influence of the prime stimulus on the reaching response, we measured the ‘path offset’ between the finger's actual position and the linear path. This is a very standard procedure when analyzing reaching trajectories (cf. [27], [44], [53] for details). As described above, this is done by first measuring the distance (D) of the straight line between the start and end points of the trajectory. Then, at each time point from target onset, the shortest distance between the tip of the index finger and the linear path is calculated in the horizontal plane (pD). Finally, the path offset value is taken as the ratio of the two distances (pD/D). The conditional mean is then calculated by averaging the path offset values at each sample for all trials within each condition, first for each subject and then across subjects.

Some researchers resample the trajectories so that every trajectory has the same number of samples [46], [54]. While this procedure allows one to average complete trajectories, it results in the loss of absolute temporal information. Because in this study we were interested in the time course of the MCE, we did not resample our trajectories. Instead, we aligned each trajectory to the target onset (time zero) and then averaged the path offset values at each time point (5 ms intervals) across trials. Because some trials conclude earlier than others, this averaging procedure begins to encounter empty cells at the end of the trajectories. To address this, we fill the empty cells with the coordinates of the last recorded sample and then we terminate the averaging procedure when more than 10% of the trials contain filled samples (this corresponded to 700 ms in the present study).

Submovements. Each fingertip trajectory was decomposed into the superposition of one or more overlapping submovements (described in more detail in [45]). The submovements were assumed to have minimum jerk velocity profiles [55]. The best selection of parameters of the submovements (their starting time, duration, and amplitude in the x and y directions) were computed using non-linear optimization in Matlab (Mathworks), using the technique described in [56], by minimizing the reconstruction error:
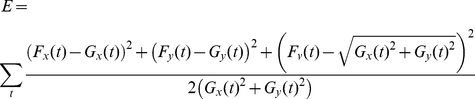
where G_x_(t) and G_y_(t) are the x and y components of the measured hand velocity, F_x_(t) and F_y_(t) are the reconstructed x and y components of the velocity. F_v_(t) is the reconstructed tangential velocity, where 
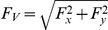
. For each reaching movement, the lowest number of submovements where the reconstructions error was less than 0.03 was selected.

It is assumed that submovements are executed in a feed-forward manner (i.e., they are planned before execution), and so the submovement amplitudes are a measure of intent, as they indicate how far the subject was planning on moving at the time of submovement onset. The cumulative submovement amplitude is the sum of the amplitude of the current and all previous submovements. Because any given trial only yields 1 to 3 submovements, we used a sliding window averaging procedure (100 ms wide) to calculate the MCE. For the graphs shown, the cumulative submovement amplitudes were calculated for each subject, by taking the average of the cumulative submovement amplitudes within a 100 ms window (50 ms before and after each time point), when there were more than 2 data points. This window moved in 20 ms steps. These cumulative amplitudes were averaged by condition for each subject and then the differences were calculated between the congruent and incongruent conditions, first for each subject and then across subjects. Cublic spline smoothing was applied to the differences to smooth the data.

#### Maintaining Experimentwise Error

As mentioned, we implemented the permutation procedure introduced by Blair and Karniski [47], who used it to carry out multiple comparisons with exact specification of experimentwise error in their analysis of ERPs. Because analyses of pointing trajectories similarly involve multiple comparisons, this procedure is ideally suited for our purposes. We point the interested reader to the original paper for a detailed explanation and rationale of this procedure. Here we only highlight the most important aspects of the implementation here. This procedure begins by assuming the null hypothesis whereby the order of the observed conditional means is arbitrary (i.e. the observed mean in Condition A is just as likely to have occurred in Condition B). Thus, to determine if the experimental manipulation had an effect, the means from each subject are systematically re-ordered, yielding 2^N^ permutations where N is the number of subjects. In our case, we tested 16 subjects, resulting in 65,536 permutations. With each permutation, a paired-sample t-test is conducted on each time point (140 samples in our experiment, for a total of 9.175 million comparisons). The maximum t-value from each permutation is saved, yielding a reference distribution of 2^N^ maximum t-values. The reference distribution associated with *t_max_* allows one to maintain experimentwise error at a prescribed level. In our case, this was done by setting the critical t-value to the value in the *t_max_* reference distribution that cut off 0.005 of each tail.

#### Prime Visibility

To calculate *d'*, we designated trials with an animal prime as the “signal present” trials and trials with a person prime as the “noise only” trials. Subjects whose *d'* score exceeded ±2.0 (N = 4) were replaced.

### Experiment 2: Letter/Number Categorization

#### Subjects

Sixteen individuals participated for course credit in an introductory psychology course. All individuals were right handed and gave informed written consent in accord with Macquarie University Human Research Ethics.

#### Materials and Design

The stimuli used in Experiment 2 are presented in Table 1. The critical targets (N = 8) were comprised of 8 different 3-item strings (4 letter strings and 4 number strings). Each letter and number appeared in one of 13 different font faces. The novel primes were novel both in terms of identity (the specific letters or numbers were not in the target set) and font (the font used for the novel primes was not repeated in the target set).

**Table 1:** Target and prime stimuli used in Experiment 2.

targets	critical	SdF	812
		bKU	931
		yRW	578
		McZ	253
	filler	EhT or aPx	790 or 464
		JnV	523
primes	repeated	EhT or aPx	790 or 464
	novel	aPx or EhT	464 or 790
	repeated/novel	eHt or ApX	790 or 464

The novel and repeated primes were counterbalanced across subjects. Novel and repeated/novel primes never appeared as targets. Novel primes were unique both at the level of abstract letter identities and physical form; repeated/novel primes mapped onto the same abstract letter identities as the repeated primes, but appeared in a novel physical form.

In each block of 120 trials, the critical targets were presented 12 times each. These 8 critical targets were preceded by each prime type (novel, repeated, repeated/novel) 4 times, twice as congruent primes and twice as incongruent primes, for a total of 96 critical (analyzed) trials per block. In this way, each prime stimulus appeared equally often across the 96 critical trials in each block. Additionally, each block included 24 filler trials. These trials consisted of 6 presentations of 4 different target stimuli, two of which were the repeated primes (the repeated letter prime and the repeated number prime). The primes on the 24 filler trials were drawn from the set of novel and repeated/novel primes and were assigned to the 4 filler targets to form congruent prime-target pairs. For example, the repeated letter prime (which appeared 6 times as a target in the filler trials) was preceded 3 times by the novel letter prime and 3 times by the repeated/novel letter prime. The same was true for the remaining filler letter target. Thus, just as the repeated primes appeared 6 times each (as targets) in the filler trials, the novel and repeated/novel primes also appeared 6 times each in these trials (but as primes). Using this design, we were able to keep the frequency of occurrence of the prime stimuli constant across the experiment with the only difference between prime types being that each repeated prime appeared as a target on 5% of the trials.

#### Procedure

**Figure 9:**
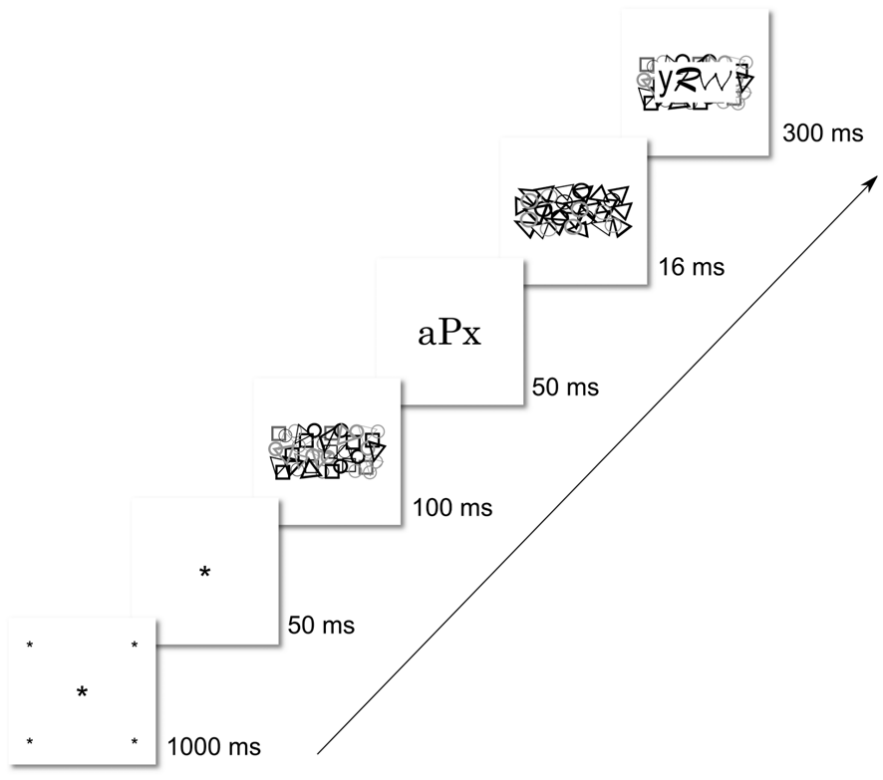
Trial Structure in Experiment 2. Here we depict a congruent trial with the novel letter string prime. The fixation frame consisted of a central point and 4 peripheral points (0.2 degrees visual angle) at the corners of an imaginary square (15 degrees). The 4 peripheral points moved smoothly towards the fixation cross over a period of 1 second whereupon the fixation point alone was presented for 50 ms followed by the forward mask for 100 ms.

The procedure was nearly identical to that of Experiment 1 with the following exceptions. First, we used a slightly different trial structure in that the stimuli were presented in black on a white background and we used a dynamic fixation frame at the onset of the trial (see Figure 9). Second, Experiment 2 included 1 practice blocks (120 trials) that were not analyzed, 2 experimental blocks (240 trials) and 1 prime detection blocks (120 trials). Subjects we given the opportunity to take a break after each run of 40 trials. Again, the trials in all blocks were identical except that in the detection blocks subjects were informed of the presence of the primes and, in this case, two possible primes appeared following target offset. Subjects indicated their response by reaching out and touching the item that they thought was the prime on the previous trial. To ensure that subjects attended to the target classification aspect of the task just as they had done in the experiment proper, we elicited prime-identification responses only on trials in which the target was correctly classified. Trials with incorrect target classifications were repeated later in the experiment to ensure an equal number of prime-identification trials across conditions.

All other aspects of the procedure were identical with Experiment 1.

#### Analysis

All aspects of the analysis were identical with Experiment 1 except for the way in which d' was calculated.

#### Prime Visibility

On each trial two items were presented and subjects were asked to touch the item that had appeared as the prime on the previous trial. Thus there was a ‘signal location’ and a ‘noise location’ on each trial. Responses were scored as hits when the signal location was chosen and false alarms when the noise location was chosen. No participant had a d' score above 2.0 and so no subjects were replaced in Experiment 2.
